# Central Pancreatectomy for Central Pancreatic Lesions: A Single-Institution Experience

**DOI:** 10.7759/cureus.16108

**Published:** 2021-07-02

**Authors:** Senthil Kumar P, Sakthivel Harikrishnan, Jeswanth Satyanesan

**Affiliations:** 1 Surgical Gastroenterology and Liver Transplant, Government Stanley Medical College, Chennai, IND

**Keywords:** central pancreatectomy, pancreas preserving resection, solid pseudopapillary neoplasm, cp, postoperative pancreatic fistula

## Abstract

Background

Pancreaticoduodenectomy and distal pancreatectomy are radical procedures for pancreatic lesions with high postoperative morbidity and mortality even in experienced hands. Central pancreatectomy is an alternative less radical procedure for centrally located pancreatic lesions that are benign or have a low malignant potential. It involves removing the central portion of the pancreas and has the advantage of preserving the pancreatic parenchyma, thereby decreasing the postoperative endocrine and exocrine insufficiencies.

Methods

We conducted a prospective study of six cases of central pancreatectomy in the Department of Surgical Gastroenterology and Liver Transplant, Government Stanley Medical College, India, between the years 2015 and 2019. All patients with lesions in the neck and proximal body of the pancreas were clinically and radiologically evaluated, and those with benign or borderline malignant lesions underwent central pancreatectomy by a standardized technique.

Results

The mean age of the patients was 27.8 years (range: 14 years - 37 years). Most of the patients were females (66.6%). The most common presenting symptom was abdominal pain, and the most common diagnosis was solid pseudopapillary neoplasm (83.3%). The mean diameter of the lesion was 6.1 cm. All patients underwent pancreaticojejunostomy of the distal stump. The median operative time and the blood loss were 310 minutes and 85 ml, respectively. Two patients developed biochemical postoperative pancreatic fistula, and in the long-term follow-up, none of them developed endocrine or exocrine insufficiency.

Conclusion

Central pancreatectomy is a safe and effective alternative for benign and low-grade lesions in the neck and body of the pancreas in which the head of the pancreas and a significant portion of the distal body and tail of the pancreas is uninvolved. Standardization of this pancreas-preserving procedure will result in better outcomes.

## Introduction

Traditional surgeries for pancreatic lesions include pancreaticoduodenectomy (PD) and distal pancreatectomy with or without splenectomy. Even in experienced hands, these surgeries carry a significant morbidity and mortality risk. These risks can be accepted if an oncological cure is the primary intent. Pancreas-preserving resections are surgeries that are aimed at preserving pancreatic parenchyma, thereby decreasing the surgical complications and the postoperative endocrine and exocrine insufficiencies in the long term. Pancreas-preserving surgeries include duodenum-sparing head resection, enucleation, and central pancreatectomy (CP) [1]. CP has become the standard surgical procedure for benign and borderline lesions in the body and neck of the pancreas. In this prospective study, we report our experience with six cases of central pancreatectomy done for benign lesions in the neck and proximal body of the pancreas.

## Materials and methods

Patients and methods

**Figure 1:**
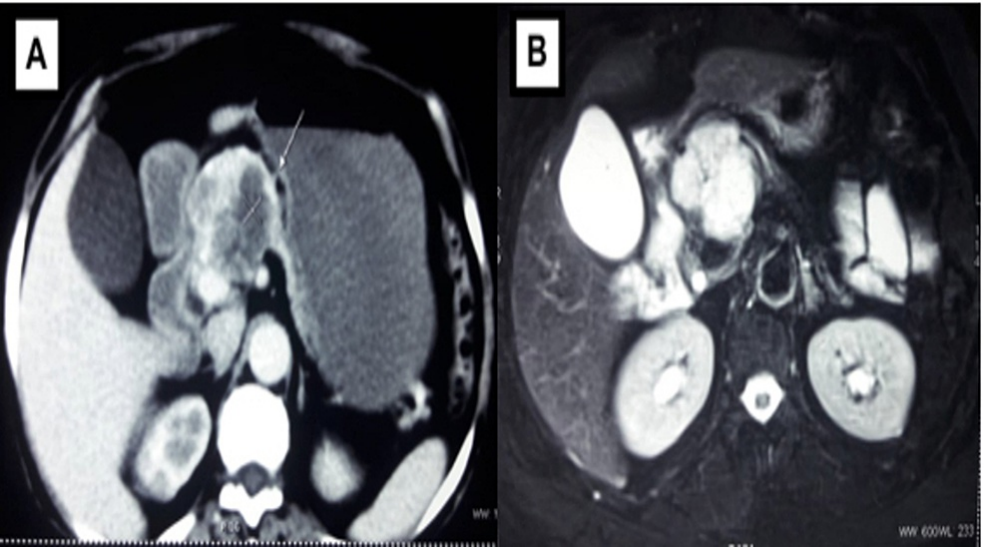
A & B: CT and MRI picture showing a heterogeneously enhancing cystic lesion in the neck of the pancreas

From January 2015 to December 2019, six cases underwent central pancreatectomy in the Department of Surgical Gastroenterology, Government Stanley Medical College, Chennai, India. All patients underwent routine blood investigations, including tumor marker (CA19-9) and imaging by pancreatic protocol CT and MRI (Figure 1).

We assessed the location and characteristics of the tumor, the relationship with the mesenteric vessels, and the remnant pancreatic volume in the imaging. Patients who had a suspicious benign lesion to the left of the gastroduodenal artery and close to the splenomesenteric confluence, with adequate remnant volume, were taken as ideal candidates for central pancreatectomy. Endoscopic ultrasound (EUS) was not included in our protocol to evaluate the cystic lesions in the pancreas due to nonavailability. Central pancreatectomy was done by the standardized technique described below.

Surgical technique

**Figure 2:**
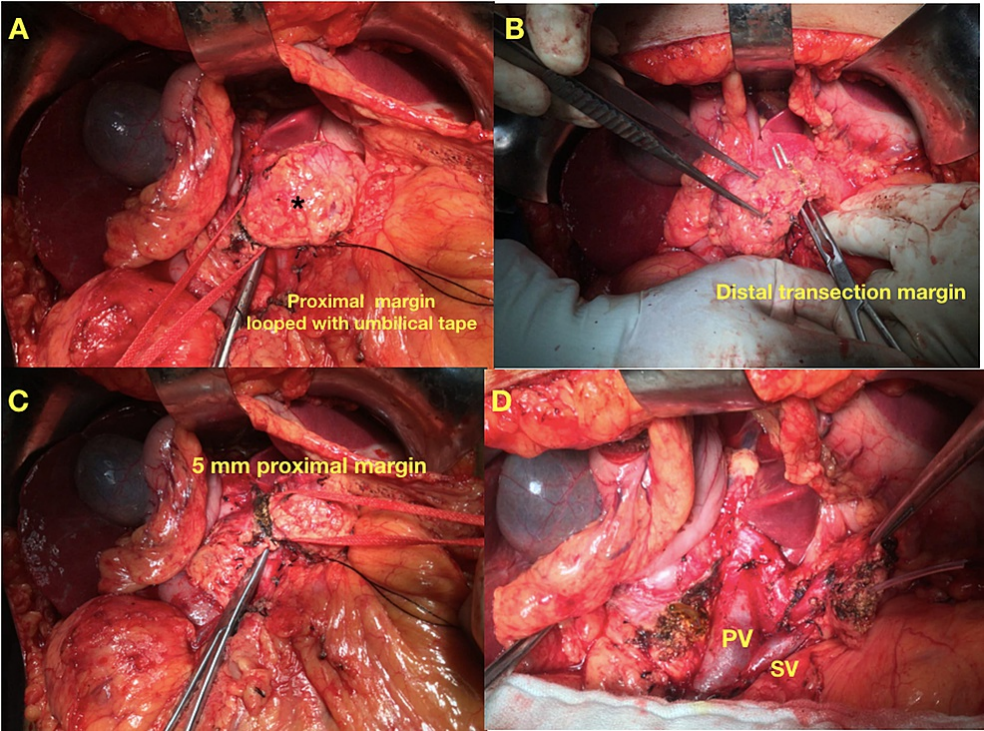
A: Lesion (*) in the proximal body of the pancreas, and after tunneling, an umbilical tape is looped around the pancreas. B: Pancreas distal to the lesion is mobilized, and an instrument is passed between the splenic vein and the posterior surface of the pancreas for distal transection. C: Proximal pancreatic parenchymal transection with 5 mm of normal pancreatic parenchyma proximal to the lesion. D: Post-resection picture showing the splenoportal axis and infant feeding tube inserted into the proximal and distal transected pancreatic duct

The abdomen was opened by a Makuuchi incision. The lesser sac was entered after dividing the gastrocolic ligament and preserving the gastroepiploic vessels. The adhesions between the posterior surface of the stomach and the pancreas were divided, and the anterior surface of the pancreas was exposed. The inferior border of the pancreas was mobilized at the level of the neck of the pancreas, and a tunnel was created between the posterior aspect of the neck of the pancreas and the portal vein. An umbilical tape was passed through this tunnel and looped around the neck of the pancreas as proximal control (Figure 2A). The splenic artery is preserved at the superior border of the pancreas. After mobilizing the inferior border of the pancreas and carefully preserving the splenic vein, distal control is taken by passing an umbilical tape 1 cm away from the lesion in the body of the pancreas (Figure 2B). The margins are marked on the pancreatic parenchyma 5 to 10 mm away from the lesion proximally and distally. Pancreatic parenchyma was transected with diathermy proximal and distal to the lesion with adequate precautions not to injure the splenoportal axis (Figure 2C). The pancreatic duct was identified in the proximal and distal cut surfaces and cannulated with an infant feeding tube (Figure 2D).

After securely closing the proximal pancreatic duct with 3-0 Prolene sutures, horizontal mattress sutures were taken on the proximal transected pancreatic surface. The distal pancreas was mobilized from the splenic vein posteriorly for 3 cm. Pancreaticojejunostomy was done end to side with the loop of jejunum passed through the retrocolic window using the Blumgart technique. Drains were placed in the lesser sac, and the Morrisons pouch and abdomen were closed in layers. At the one-year follow-up, the endocrine function was assessed by measuring fasting and postprandial blood sugars. Patients who had weight loss, diarrhea, and fatty stools were considered to have exocrine insufficiency.

## Results

A total of six patients underwent a central pancreatectomy. The mean patient age was 27.8 years (range: 14 years - 37 years) and most of them are females (66.6%). The most common presenting symptom was abdominal pain. All patients had normal CA19-9 levels. One patient was referred to us after EUS biopsy showed evidence of a solid pseudopapillary neoplasm. The most common indication was a solid pseudopapillary neoplasm (83.3%) followed by mucinous cystadenoma (16.7%). The mean tumor size was 6.1 cm. All patients underwent pancreaticojejunostomy. The median operative time and blood loss were 310 minutes and 85 ml, respectively. Two patients (33%) developed a biochemical postoperative pancreatic fistula. The mean duration of hospital stay was 12 days. During the one-year follow-up, none of the patients developed impaired glucose tolerance or pancreatic insufficiency. The clinical characteristics of the patients are summarized in Table 1.

**Table 1:** Clinical data of patients with benign neck and body lesions who underwent central pancreatectomy *POPF: Postoperative Pancreatic Fistula (ISGPF 2016 Definition)

Serial No.	Age /Sex	Presentation	Imaging	Histopathology	POPF*	Endocrine Insufficiency (1- year follow-up)	Exocrine Insufficiency (1-year follow-up)
1.	14/Female	Pain abdomen	5x5 cm heterogeneously enhancing solid cystic lesion in the neck	Solid pseudopapillary neoplasm	Biochemical	No	No
2.	24/Female	Incidentally detected during antenatal scans	4x3 cm heterogeneously enhancing solid cystic lesion in the neck	Solid pseudopapillary neoplasm	Biochemical	No	No
3.	36/ Male	Pain abdomen and elevated alkaline phosphatase	7x7 cm heterogeneously enhancing solid cystic lesion in the neck and the body of the pancreas	Solid pseudopapillary neoplasm	Nil	No	No
4.	29/Female	Pain abdomen	11x9 cm solid cystic lesion in the neck and body	Solid pseudopapillary neoplasm	Nil	No	No
5.	27/ Male	Pain abdomen	5x4 cm solid cystic lesion in the neck and the body	Solid pseudopapillary neoplasm	Nil	No	No
6.	37/Female	Pain abdomen	5x4 cm cystic lesion in the body of the pancreas	Mucinous cystadenoma	Nil	No	No

## Discussion

The standard surgical procedure for benign or borderline lesions in the neck and proximal body of the pancreas is Whipple’s procedure or distal pancreatectomy. Though the mortality associated with these procedures has decreased, the morbidity is still significant for them to be considered as an option for nonmalignant lesions of the pancreas. The loss of normal pancreatic parenchyma in these radical procedures also leads to long-term functional consequences like endocrine and exocrine insufficiency [2].

A central pancreatectomy is a pancreas-preserving option for benign and borderline lesions in the neck and the proximal body of the pancreas with significantly low morbidity and nil mortality [3-4]. It cannot be done for proven malignant lesions in the neck and body of the pancreas due to the lack of oncological radicality. Hence, before contemplating central pancreatectomy for all patients in our series, the clinical, radiological, and intraoperative findings are analyzed to rule out a malignant lesion of the pancreas.

The most common indications for central pancreatectomy are pancreatic neuroendocrine tumors (pNET), followed by serous cystadenomas, intraductal papillary mucinous neoplasm (IPMN), mucinous cystadenoma, and solid pseudopapillary neoplasm (SPN) [5]. The most common indication in our series was SPN.

The main Achilles heel of central pancreatectomy is the risk of a pancreatic fistula from two pancreatic cut surfaces and the pancreaticoenteric anastomosis. This risk is further augmented by the presence of a soft pancreas and a non-dilated pancreatic duct. The rate of pancreatic fistula after pancreaticoduodenectomy and distal pancreatectomy is 5%-25% [5]. Though the rate of pancreatic fistula after CP is high (26%-35%), the risk of the clinically relevant fistula is less in most of the published CP series [6-7]. Exceptionally, a recent case-control study has shown that CP has a more clinically relevant POPF and poorer preservation of endocrine and exocrine function when compared with DP. But it concluded that CP is better than PD in terms of better exocrine function and preservation of remnant pancreatic volume. There is no difference in POPF between pancreaticogastrostomy and pancreaticojejunostomy. Patients who underwent pancreaticogastrostomy were found to have more atrophic pancreatic tail changes [8]. Central pancreatectomy has significantly better weight gain at two years after surgery when compared with distal pancreatectomy due to a better exocrine function in the former [9]. The postoperative glycemic control with central pancreatectomy fares better than the standard pancreatic resections [10]. All the patients in our series underwent a standardized end-to-side pancreaticojejunal anastomosis and didn’t have any clinically relevant POPF, and in the one-year follow-up, none of them had exocrine or endocrine insufficiency.

The following are the main advantages of central pancreatectomy over the standard pancreatic resections based on the available literature: 1. Whipple's procedure and distal pancreatectomy is associated with endocrine insufficiencies in 15% and 50%-60 % of the patients, respectively. A meta-analysis has shown that central pancreatectomy significantly reduces the risk of the onset of diabetes when compared to distal pancreatectomy (5.5% vs. 23.6%). The preservation of exocrine function was borderline significant when compared to the distal pancreatectomy [11]. 2. The gastrointestinal and biliary continuity is unaltered, thereby avoiding metabolic and nutritional complications. 3. The chances of preserving the spleen are more when compared to distal pancreatectomy. 4. Though the occurrence of the pancreatic fistula is high after central pancreatectomy, most of them are biochemical leaks or grade B fistulas, which can be managed conservatively or by percutaneous drainage [11-12].

Central pancreatectomy can also be done by the laparoscopic approach and robotic-assisted laparoscopic approach with comparable fistula rates to open pancreaticogastrostomy is the preferred reconstruction technique after the laparoscopic approach because of the anatomic proximity and technical ease [13-15]. A large case series of 100 patients who underwent central pancreatectomy showed that the learning curve improved the operative time and the blood loss but the clinically relevant POPF rates (32%) and the biochemical leak rate (14%) hasn’t shown much improvement [16]. Both these approaches reduce postoperative morbidity when compared with the open procedure.

Distal pancreatectomy (DP) is the other most commonly considered surgical option for lesions in the neck and the body of the pancreas. Several studies have compared CP vs. DP as a surgical option for benign lesions in the proximal body of the pancreas. The study by Ocuin et al. showed that the central pancreatectomy patients had a significantly higher risk of complications than extended left pancreatectomy. There was no significant risk of major complications or hospital stay in the CP group. Patients who had undergone extended left pancreatectomy had a higher incidence of clinically significant new-onset diabetes [17]. The meta-analysis of Iacono et al. comparing central pancreatectomy with distal pancreatectomy concluded that though the risk of overall morbidity and fistula was high, the risk of reoperation and long-term endocrine failure showed a clinically significant reduction [11]. A multicenter study in which our center took part in India on cystic tumors of the pancreas reported that the most common cystic tumor was solid pseudopapillary epithelial neoplasm (28%) and central pancreatectomy was done in only 8.5% of the cases [18]. In our center, we select patients with definitely benign lesions in the body of the pancreas based on imaging, clinical parameters, and intraoperatively if sufficient remnant pancreatic head volume and tail volume is present, we proceed with CP. Patients who have insufficient pancreatic tail volume undergo distal pancreatectomy.

The main limitation of the study is the small sample size because of the rare occurrence of benign lesions in the body of the pancreas suitable for central pancreatectomy. There are no randomized trials to compare the parenchyma-preserving CP with radical pancreatic resections like distal pancreatectomy or pancreaticoduodenectomy. The published literature includes mostly large case series, which clearly shows better long-term endocrine and exocrine functions with CP. Since we didn't take a preoperative biopsy to objectively rule out a malignant lesion, central pancreatectomy would be oncologically inappropriate if the lesion turns out to be malignant. The other drawback is, we didn’t objectively measure the pancreatic exocrine insufficiency in follow-up. Patients who had steatorrhoea and significant weight loss were considered to have exocrine insufficiency in our series.

## Conclusions

Central pancreatectomy is a safe and effective alternative for benign and low-grade lesions in the neck and the proximal body of the pancreas in which the head of the pancreas and a significant portion of the distal body and tail of the pancreas is uninvolved. Theoretically, though the risk of the pancreatic fistula is high, it is usually easily manageable. Standardization of this surgical procedure might lead to better outcomes and quality of life in the long term.
